# Platform evaluation and lessons learned with IT-supported patient recruitment

**DOI:** 10.1038/s41746-026-03366-y

**Published:** 2026-10-30

**Authors:** Cosima Strantz, Romina Blasini, Andreas Kühn, Max Behrens, Alexandra Stein, Jakub Lidke, Hans-Ulrich Prokosch, Martin Boeker, Christian Gulden

**Affiliations:** 1 Friedrich-Alexander-Universität Erlangen-Nürnberg, Medical Informatics, Erlangen, Germany; 2 Data Integration Center, Institute of Medical Informatics, Justus Liebig University, Giessen, Germany; 3 Department of Neurology, University Hospital Carl Gustav Carus at Technical University of Dresden, Dresden, Germany; 4 Institute of Medical Biometry and Statistics, Medical Faculty and Medical Center, University of Freiburg, Freiburg, Germany; 5 Institute for Community Medicine, Section Epidemiology of Health Care and Community Health, University Medicine Greifswald, Greifswald, Germany; 6 Data Integration Center, Medical Faculty, Philipps University Marburg, Marburg, Germany; 7 School of Medicine and Health, Institute of Artificial Intelligence and Informatics in Medicine, Technical University of Munich, Munich, Germany

**Keywords:** Clinical trials, Technology

## Abstract

Recruiting participants for clinical trials is a challenge, often limited by inefficient methods for identifying eligible candidates. Clinical trial recruitment support systems (CTRSS) can enhance recruitment efficiency by leveraging data from electronic health records (EHR). We evaluated two CTRSS—recruIT and KAS+—to quantify their real-world impact on enrollment. Within the German MIRACUM consortium, we conducted a multicenter segmented interrupted time-series (ITS) evaluation across six university hospitals. Twenty-nine trials contributed 814 observation weeks spanning a control phase (usual practice) and an intervention phase (CTRSS active). Weekly enrollments were compared using segmented negative binomial mixed models estimating level and slope changes at implementation, complemented by Wilcoxon signed-rank tests. A criteria revision covariate captured iterative refinement of computable cohort definitions. In pooled ITS analyses, we observed neither a statistically significant immediate level change nor a statistically significant post-implementation slope change in weekly enrollments. Effects were highly heterogeneous across trials and specialties; implemented eligibility logic most often relied on diagnosis, age, and visit data, while laboratory values and structured observations were seldom available. Higher values of the criteria revision covariate were associated with higher recruitment in some settings, with the most exploratory signal observed at the single KAS+ site. Despite the potential benefits of CTRSS, their effectiveness depends on careful customization and implementation. Overall, our findings suggest that CTRSS impact is context-dependent and that measurable benefit is more likely driven by iterative computable phenotyping and local workflow fit rather than a simple “switch-on” effect. Context-specific adaptation and continual revision of eligibility logic appear critical to realizing benefit. Future studies should reduce trial heterogeneity, strengthen compliance incentives, and employ granular outcomes, such as identification-to-enrollment latency, to better capture workflow-mediated effects.

## Introduction

Clinical trials are biomedical research experiments involving human subjects that advance medical science, represent a valuable step toward new treatments, and provide the most reliable method to establish causal evidence between treatment and outcome in clinical practice^1^. Yet successful participant recruitment is a major challenge in conducting clinical trials, as inadequate enrollment can delay trial timelines, increase costs, and compromise the validity of study results. This challenge is even more pronounced in translational research, where timely enrollment of suitable patients is often a key barrier to efficient trial execution and scientific progress^2,3^. Despite the abundance of data stored in electronic health records (EHRs), identifying eligible individuals remains difficult due to a lack of efficient and widely implemented methods^4^. While several tools have been developed, their use in routine practice is still limited, and real-world evaluations remain scarce.

In current practice, clinical trial staff usually screen patients manually by reviewing EHRs to extract demographic and clinical data, which are then compared with predefined eligibility criteria^5,6^. This process is time-consuming and labor-intensive, particularly given the volume of data recorded in EHRs and the need for timely recruitment^6,7^. There are indications in the literature that the number of study participants can be increased by using electronic clinical trial recruitment support systems (CTRSS)^8–10^. They address this challenge by facilitating the identification of trial participants through the secondary use of data routinely collected during patient care^11^. To implement a CTRSS, EHR data and eligibility criteria (ECs) must be available in a computerized format to compare the two and generate a list of potential subjects^12,13^. The current literature frequently describes CTRSSs; however, most reports focus on the development and application of CTRSSs for specific trial types or single departments^9,11,14,15^.

Within the German Medical Informatics Initiative (MII), MIRACUM is one of four consortia funded by the Federal Ministry of Education and Research (BMBF, now BMFTR) that established Data Integration Centers (DIC) at each of the ten MIRACUM university hospitals to transform routine patient data into harmonized research repositories^16^. Based on this technical infrastructure, two CTRSS—recruIT and KAS+—were implemented and used for the first time in this study. RecruIT is a tool based on the Observational Medical Outcomes Partnership Common Data Model (OMOP CDM) and Health Level 7 Fast Healthcare Interoperability Resources (HL7 FHIR) to support interoperable, automated patient pre-screening^17^. KAS+ is an EHR-integrated tool that supports clinical trial pre-screening with customizable alerts and pseudonymized screening lists. Both systems are functionally similar and use structured routine care data to automatically identify potential trial participants based on predefined eligibility criteria^18^.

The objective of this study is to assess whether the recruitment tools recruIT and KAS+ measurably affect clinical trial recruitment—either by increasing the number of enrolled participants or by accelerating the recruitment process. The hypothesis tested is that the use of an IT-supported recruitment solution positively impacts recruitment success in university hospital settings.

Hereafter, we refer to the evaluation as the “study” and to the clinical trials as “trials”.

## Results

### Trials

**Fig. 1:**
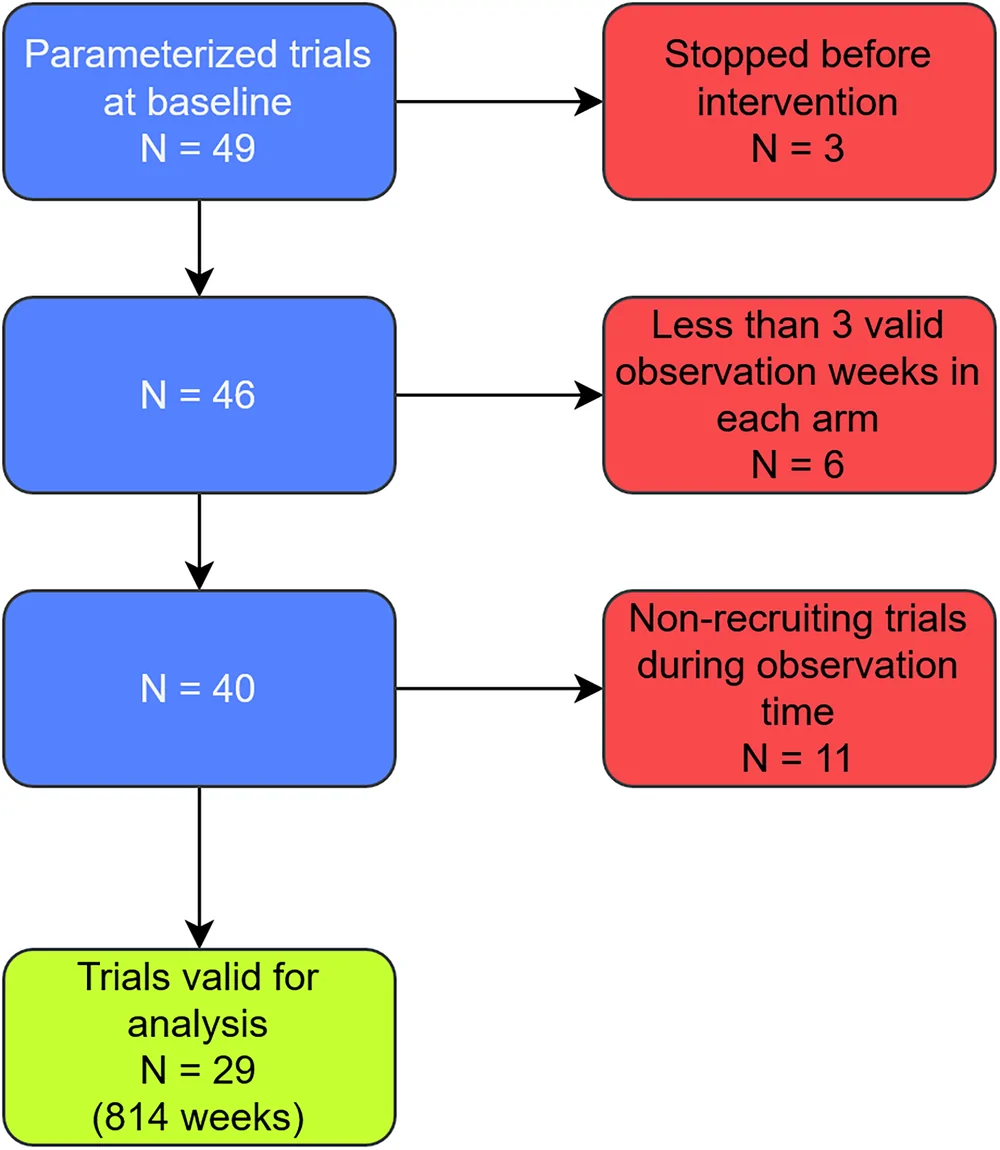
A total of 49 trials around all sites could be initially included in the evaluation study. After exclusion of trials that did not fulfill the eligibility criteria, 29 evaluable trials remained.

As shown in Fig. 1, a baseline cohort of *N* = 49 trials across all sites was analyzed. The number of initially included trials per site ranged from 4 to 15. Three trials were excluded due to premature termination, 6 due to insufficient valid observation weeks (at least 3 weeks in each control and intervention phase), and eleven due to the absence of any recruitment during the observation period. Ultimately, *N* = 29 trials, with 814 observation weeks, were included in the analysis.

The selected trials covered a broad range of specialties, with 16 in neurology, 6 in internal medicine, 2 in urology, and one each in dermatology, otorhinolaryngology (ENT), intensive care, neurosurgery, and pediatrics. 14 trials involved patient interventions, 15 were observational, 14 were randomized, and 11 were blinded.

**Fig. 2:**
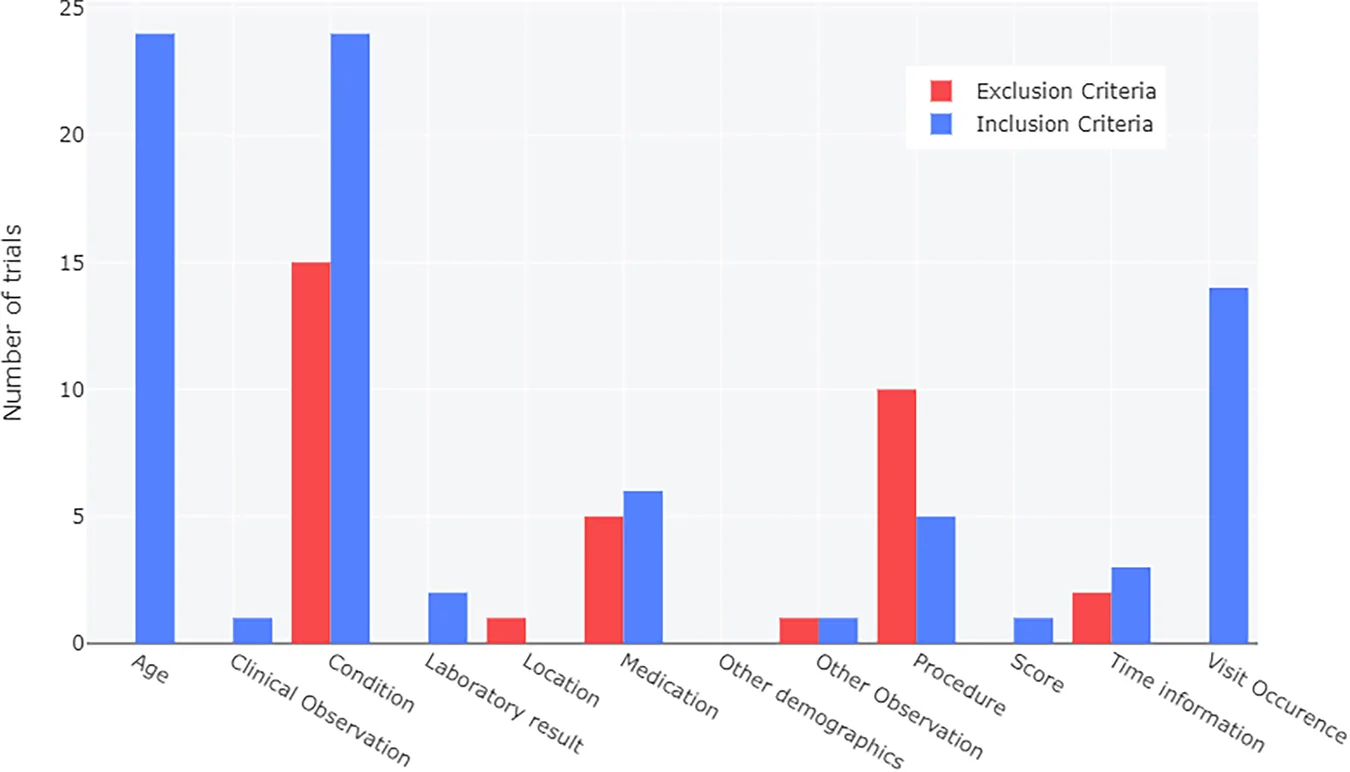
Comparison of the different categories of inclusion criteria with the number of studies in which they occur. The values are divided into inclusion and exclusion criteria.

Analysis of the eligibility criteria revealed that “condition” and “age” criteria were applied in 82.7% (*N* = 24) of the trials, while patient visit information featured in 48.3% (*N* = 14) of the cases. In contrast, laboratory values and clinical observations were used in only two trials each. Figure 2 shows the distribution of inclusion and exclusion criteria types.

### Recruitment patterns across study phases

**Fig. 3:**
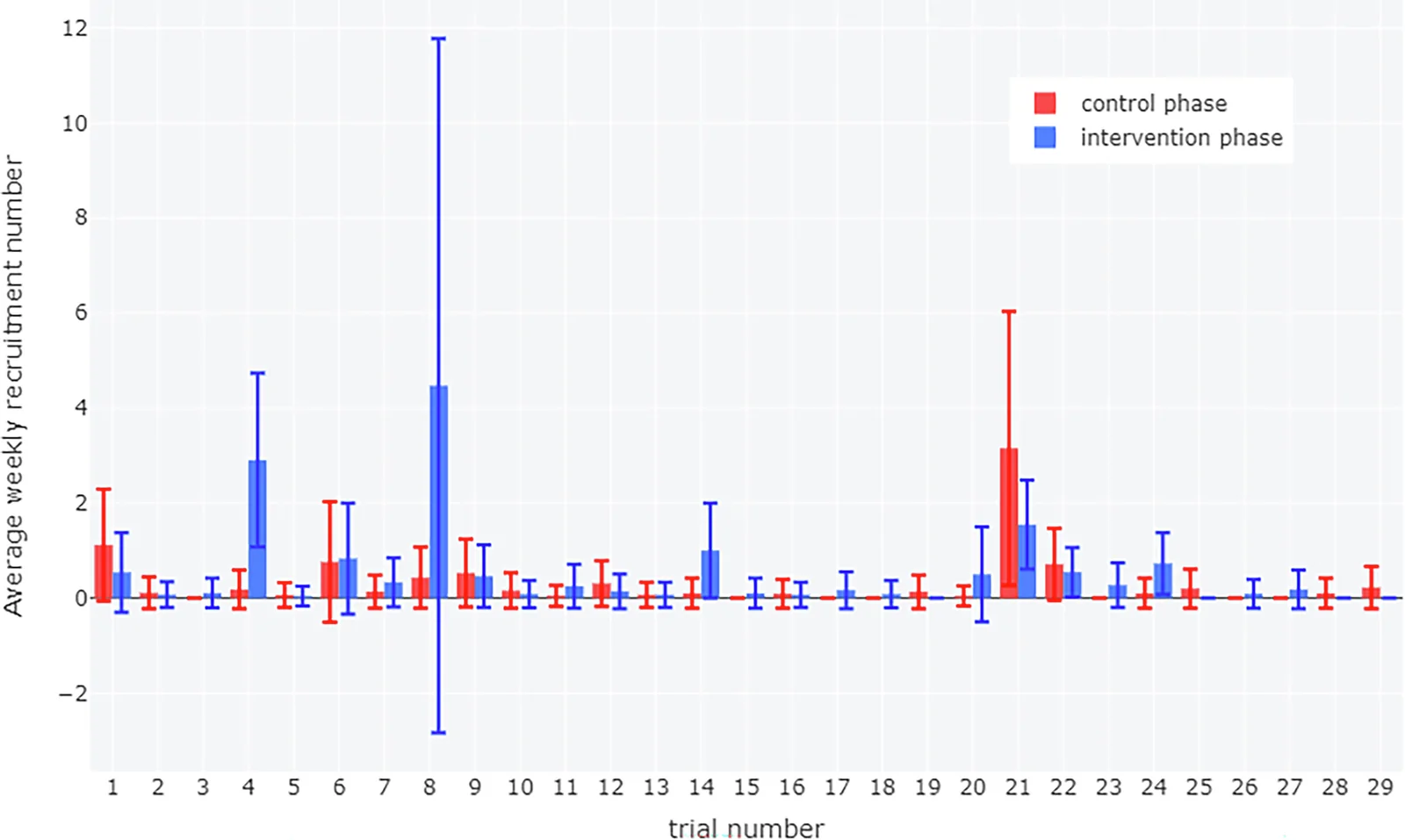
List of the weekly recruitments of all individual trials. A distinction was made between the intervention phase (blue) and the control phase (red). The error bars show the standard deviation from the mean value.

As shown in Fig. 3, the comparison of the average weekly recruitment numbers (weekly recruitments) between control and intervention phases shows that the impact of the intervention on the weekly recruitments varies across the observed trials. For example, trials 4 and 8 showed marked increases in recruitment, whereas trials 1 and 21 experienced declines. The considerable standard deviation (SD) further shows substantial variability in recruitment rates over the trial weeks.

The study aimed to evaluate the impact of the intervention by comparing the metrics from the control and intervention phases. Table 1 presents the summary statistics for the weekly recruitments observed during the control and intervention phases. On average, weekly recruitment increased from 0.30 (SD = 0.61) in the control phase to 0.54 (SD = 0.97) in the intervention phase. The highest trial-level mean weekly recruitment was also higher during the intervention phase than during the control phase (4.47 vs. 3.15).

**Table 1:** Summary statistics of average weekly recruitment rates per trial during control and intervention phases

Statistics	control phase	intervention phase
**Mean**	0.3	0.54
**Standard deviation (SD)**	0.61	0.97
**Minimum**	0	0
**Maximum**	3.15	4.47

Values represent the mean number of participants recruited per week, averaged over the respective observation periods for each trial.

Decimal values (e.g., 3.15 or 4.47) result from this averaging and do not refer to individual weekly patient counts.

To assess the significance of the differences between the weekly recruitments in the control and intervention phases, a Wilcoxon signed-rank test was performed. The results of this test are summarized in Table 2. The test yielded a W statistic of 153, with a *z* value of −1.14. The *p*-value associated with this test was 0.26, indicating that the observed differences between the control and intervention phases are not statistically significant at the conventional alpha level of 0.05. Additionally, the effect size (*r*) was calculated to be 0.22, which is considered a small effect size.

**Table 2:** Wilcoxon signed-rank test results for the weekly recruitments

**Comparison**	***W***	***z***	***p***	***r***
**intervention phase-control phase**	153	-1.14	0.26	0.22

### Heterogeneity of trials

We collected data on the weekly recruitments and the intervention effect, with the y-axis ranging from −1 to +1 to represent the magnitude and direction of the effect.

**Fig. 4:**
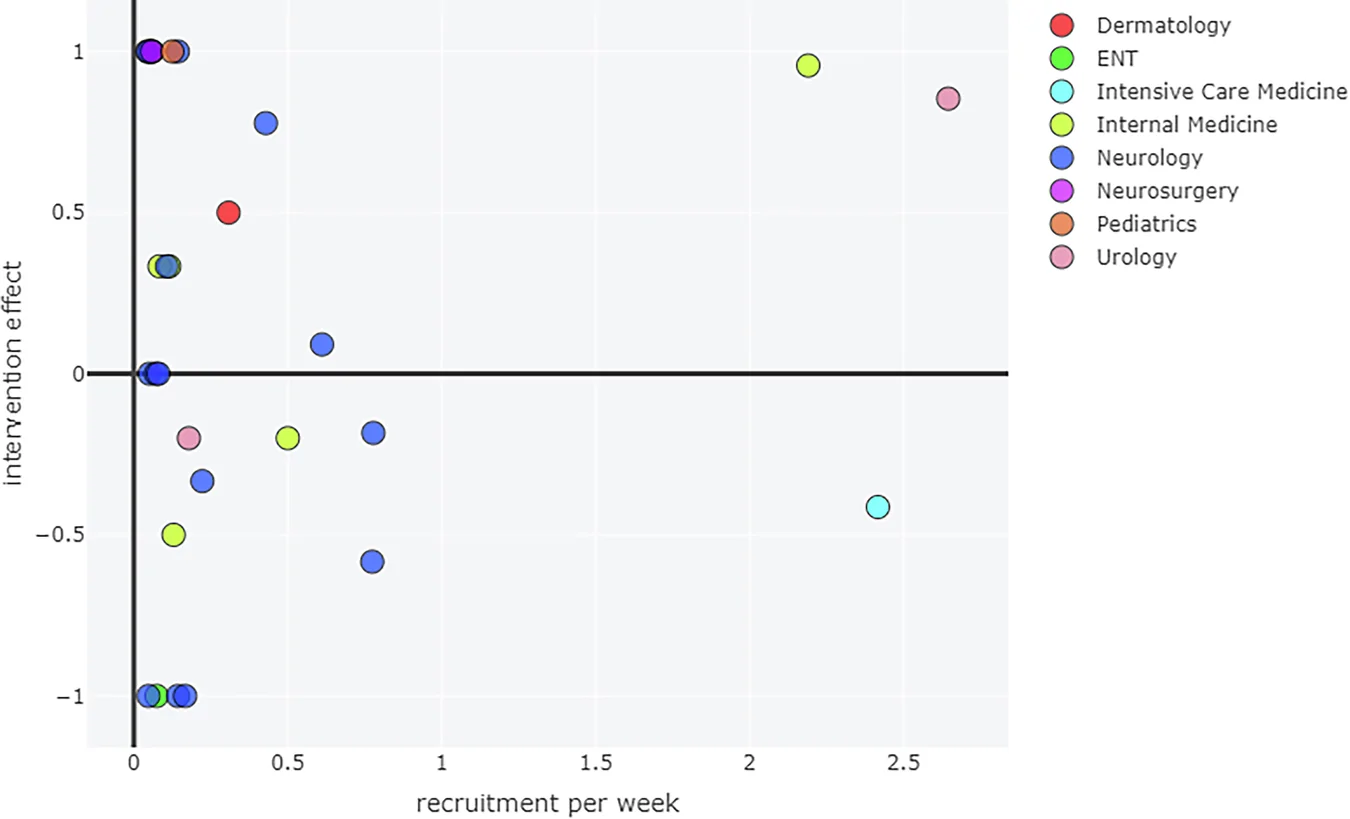
Intervention effect of all medical departments. Presentation of the intervention effect per study in relation to the average weekly recruitment numbers; the colors indicate the discipline of the corresponding trials. The x-axis represents mean weekly recruitment numbers, y-axis the intervention effect and each point one clinical trial.

In Fig. 4 the x-axis represents the weekly recruitments, and the y-axis represents the intervention effect, defined as the difference in weekly recruitments between the control and intervention phases.

**Table:** 

${\overline{rec}}$	Mean weekly recruitment number of a trial	
${\overline{rec}}_{{control}}$	Mean weekly recruitment number of a trial in observation phase	
${\overline{rec}}_{intervention}$	Mean weekly recruitment number of a trial in intervention phase	
${intervention} \, {effect} ={{\overline{rec}}_{intervention}-{\overline{rec}}_{control}}$		>0: higher recruitment in intervention phase = 0: no difference between phases < 0: lower recruitment in intervention phase

If the weekly recruitments are higher in the intervention phase than in the control phase, the intervention effect is positive. Conversely, if the weekly recruitments are lower in the intervention phase than in the control phase, the intervention effect is negative. Each point on the plot corresponds to one clinical trial. Different colors denote different clinical disciplines. The position of each point indicates the weekly recruitments and the intervention effect for the respective clinical trial. Points above the x-axis indicate trials in which the intervention had a positive effect on recruitment rates, while points below the x-axis indicate trials in which the intervention had a negative effect, as observed in 11 trials. The spread of points across the plot reflects the variation in weekly recruitments and intervention effects across clinical disciplines.

### Mixed model analysis

Table 3 presents the incidence rate ratios (IRR) with two-tailed 95% confidence intervals (CI) and p-values for the fixed effects from the negative binomial mixed models. Model A (crude) included only the intervention, while Model B additionally included the revision of the cohort definition in the CTRSS (criteria revision). The number of criteria revisions was unevenly distributed across trials and sites (median [IQR] maximum revision per trial: 0 [0–1]; range 0–4; Supplementary Table S1). Most updates occurred during the early intervention phase (Supplementary Table S1). Across all study sites, the intervention effect was not statistically significant in either Model A (IRR 1.731, 95% CI 0.922 to 3.251; *p* = 0.088) or Model B (IRR 1.344, 95% CI 0.710 to 2.544; *p* = 0.363). In Model B, the criteria revision covariate showed a statistically significant association with recruitment across all sites (IRR 1.601, 95% CI 1.143 to 2.242; *p* = 0.006). Model-based marginal predictions illustrating the expected weekly enrollments across criteria revision values (v0–v4) are provided in Supplementary Table S2.

**Table 3:** Incidence Rate Ratios (with 95% confidence interval) for intervention (crude model) and intervention and criteria revision effect on weekly

Model	study sites (*n*)	trials (*n*)	weeks (*n*)	Parameter	Incidence Rate Ratio (95% confidence interval)	*P*-value
**All study sites**						
A-crude	6	29	814	intervention	1.731 (0.922 to 3.251)	0.088
B-with criteria revision	6	29	814	intervention	1.344 (0.71 to 2.544)	0.363
				criteria revision	1.601 (1.143 to 2.242)	0.006
**recruIT definitions**						
A-crude	5	25	620	intervention	1.597 (0.837 to 3.048)	0.156
B-with criteria revision	5	25	620	intervention	1.504 (0.75 to 3.015)	0.251
				criteria revision	1.161 (0.627 to 2.152)	0.635
**KAS+ definitions**						
A-crude	1	4	194	intervention	2.847 (0.403 to 20.142)	0.294
B-with criteria revision	1	4	194	intervention	1.23 (0.186 to 8.122)	0.829
				criteria revision	2.151 (1.784 to 2.593)	< 0.001

A generalized mixed model with negative binomial distribution was used with each trial as a cluster for random intercept and random slope for intervention. Model A only used intervention (yes/no) as fixed effect, model B used intervention and criteria revision of the cohort definition as 2 fixed effects. First, all study sites and second, study sites with the recruIT definition tool as well as the KAS+ definition tool were analyzed.

In exploratory analyses stratified by CTRSS type, sites using recruIT showed no statistically significant effects for either the intervention (Model A: IRR 1.597, 95% CI 0.837 to 3.048; *p* = 0.156; Model B: IRR 1.504, 95% CI 0.75 to 3.015; *p* = 0.251) or criteria revision (IRR 1.161, 95% CI 0.627 to 2.152; *p* = 0.635). At the single site using KAS+, the intervention effect was not statistically significant in either Model A (IRR 2.847, 95% CI 0.403 to 20.142; *p* = 0.294) or Model B (IRR 1.23, 95% CI 0.186 to 8.122; *p* = 0.829), whereas the criteria revision effect was statistically significant (IRR 2.151, 95% CI 1.784 to 2.593; *p* < 0.001). All models showed good calibration, and residuals did not show significant dispersion.

**Fig. 5:**
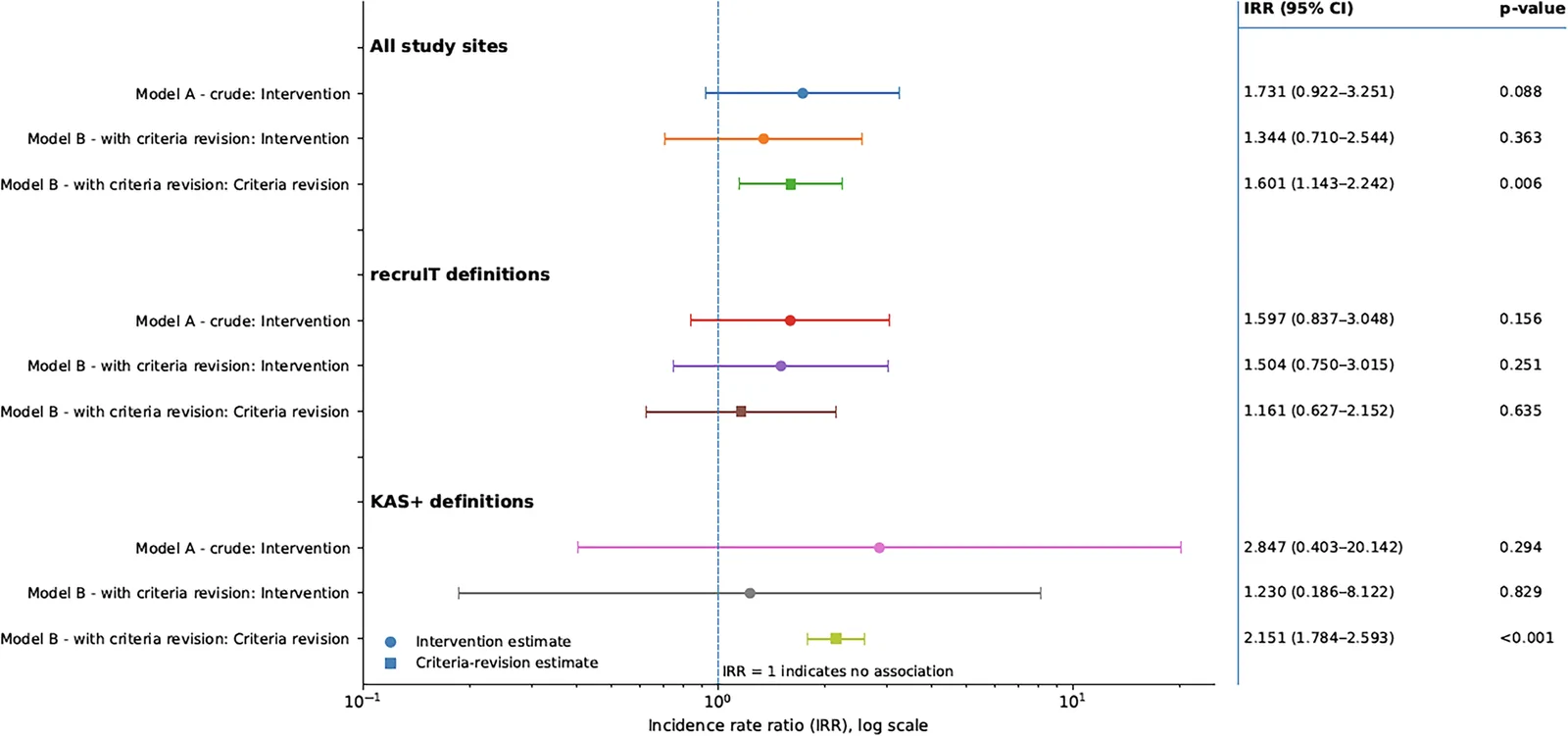
Forest plot of Model A and Model B estimates from the negative binomial mixed models. The figure shows incidence rate ratios (IRR) with 95% confidence intervals for the intervention and criteria-revision estimates reported in Table 3, stratified by all study sites, recruIT definitions, and KAS+ definitions. Model A includes the intervention indicator only, whereas Model B additionally includes criteria revision/version as an ordinal covariate. The vertical dashed line at IRR = 1 indicates no association. Intervention estimates were not statistically significant in any group. Criteria revision was significantly associated with higher weekly recruitment across all study sites and at the single KAS+ site, but not in the recruIT subgroup.

To support interpretation of Table 3, Fig. 5 presents the corresponding forest plot of the Model A and Model B estimates. Consistent with Table 3, all intervention confidence intervals crossed the null value of IRR = 1, indicating no statistically significant intervention effect in the pooled analysis or in the CTRSS-specific subgroup analyses. In contrast, the criteria-revision estimate in Model B was statistically significant across all study sites and at the single KAS+ site, whereas the corresponding estimate for recruIT definitions was not statistically significant.

Given the heterogeneous model patterns across CTRSS type and study sites, we further assessed whether the intervention effect varied according to trial- and eligibility-related characteristics. To explore heterogeneity, we conducted exploratory interaction models testing effect modification by eligibility-criteria complexity (number of inclusion/exclusion/total criteria) and exploratory specialty stratification. Results are summarized in Supplementary Table S3.

### Segmented interrupted time-series analysis

**Fig. 6:**
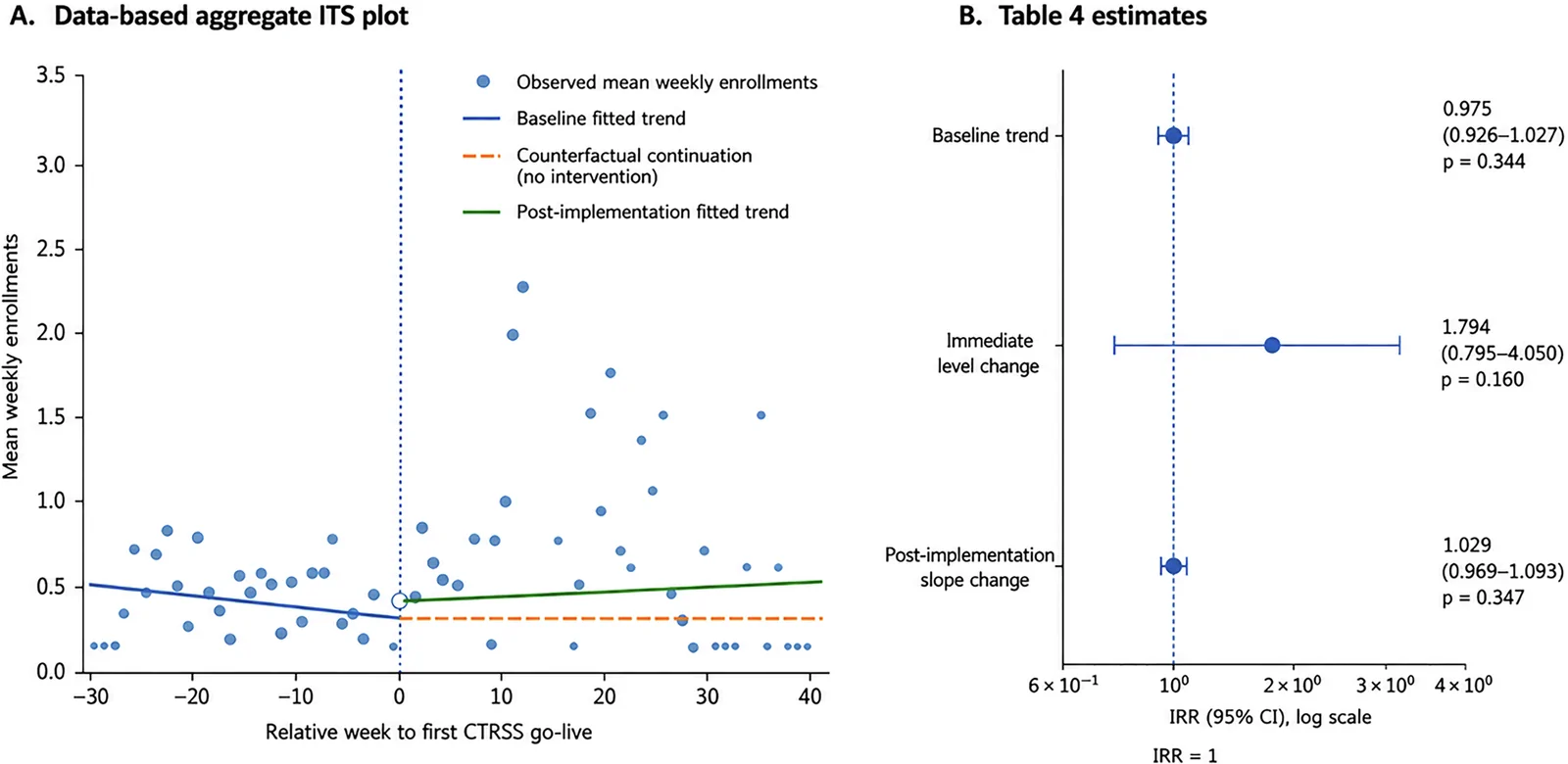
Data-based segmented interrupted time-series visualization and model estimates. **A** Shows observed mean weekly enrollments by relative week to the first CTRSS go-live, with week 0 indicating implementation. The lines show the fitted baseline trend, the counterfactual continuation of the pre-implementation trend, and the post-implementation fitted trend based on the segmented ITS model. **B** Shows the corresponding fixed-effect estimates from Table 4 as incidence rate ratios (IRR) with 95% confidence intervals. The vertical dashed line at IRR = 1 indicates no association. Neither the immediate level change at implementation nor the post-implementation slope change was statistically significant.

In the segmented ITS model, we did not observe a statistically significant immediate level change at implementation (post: IRR 1.794, 95% CI 0.795–4.050; *p* = 0.160) or a statistically significant change in slope after implementation (time_after: IRR 1.029, 95% CI 0.969–1.093; *p* = 0.347). The baseline time trend was also not statistically significant (time: IRR 0.975, 95% CI 0.926–1.027; *p* = 0.344). Figure 6 visualizes the aggregate ITS pattern using observed mean weekly enrollments aligned to the first CTRSS go-live and displays the corresponding fixed-effect estimates, illustrating that the confidence intervals for both the immediate level change and the slope change include IRR = 1 (Table 4 and Fig. 6). In a sensitivity analysis restricted to the 26 single-switch trials (719 trial-weeks), neither the immediate level change nor the post-implementation slope change was statistically significant, and the results were consistent with the primary segmented ITS analysis, see Supplementary Table S6.

**Table 4:** Segmented interrupted time-series analysis of weekly enrollments

Model component	Interpretation	IRR	95% CI	*p*-value
**time**	Baseline time trend before implementation	0.975	0.926–1.027	0.344
**post**	Immediate level change at CTRSS implementation	1.794	0.795–4.050	0.160
**time_after**	Change in slope after CTRSS implementation	1.029	0.969–1.093	0.347

Fixed-effect estimates from the segmented negative binomial interrupted time-series model are reported as incidence rate ratios (IRR) with two-sided 95% confidence intervals and *p*-values. The model includes the baseline time trend (time), the immediate level change at CTRSS implementation (post), and the change in slope after implementation (time_after). Trial-level clustering was accounted for as described in the “Methods”.

### Exploratory lag and implementation-intensity analyses

To assess potential delayed associations, we fitted exploratory models including 1–4-week lags of the intervention indicator while adjusting for the concurrent intervention term. No consistent lagged intervention effect was observed (lag 1: IRR 0.794, *p* = 0.596; lag 2: IRR 1.167, *p* = 0.723; lag 3: IRR 1.218, *p* = 0.654; lag 4: IRR 1.233, *p* = 0.616). Similarly, lagged criteria revision terms were not statistically significant for lags 1–3. The four-week lag of criteria revision approached, but did not reach, statistical significance (IRR 0.842, 95% CI 0.705–1.004; *p* = 0.056), details in Supplementary Table S4a.

We additionally stratified trials by implementation intensity (maximum number of criteria revisions per trial). Trials without any documented updates (maximum number of criteria revisions=0; 20 trials/538 weeks) showed no significant intervention effect (IRR 1.487, 95% CI 0.607–3.643; *p* = 0.385). In trials with ≥1 update (maximum number of criteria revisions≥1; 9 trials/276 weeks), the crude intervention effect was also non-significant (IRR 1.606, 95% CI 0.358–7.193; *p* = 0.536); when modeling intervention jointly with number of criteria revisions, revision was strongly associated with recruitment (IRR 2.302, 95% CI 1.948–2.720; *p* < 0.001), while the intervention term was attenuated (IRR 0.413, 95% CI 0.178–0.957; *p* = 0.039). Results are reported in Supplementary Table S4b.

### Sensitivity analysis

As a sensitivity analysis, we repeated Models A and B including all observation weeks and adjusted for inactive weeks using an indicator (inactive_week). The intervention effect remained non-significant (Model A: IRR 1.622, 95% CI 0.796–3.305; *p* = 0.183; Model B: IRR 1.252, 95% CI 0.579–2.706; *p* = 0.568). Inactive weeks were associated with lower weekly enrollments (Model A: IRR 0.473, 95% CI 0.280–0.802; *p* = 0.005; Model B: IRR 0.493, 95% CI 0.281–0.865; *p* = 0.014). The criteria revision term remained positive but was not statistically significant in this sensitivity model (IRR 1.506, 95% CI 0.944–2.403; *p* = 0.086). Results are provided in Supplementary Table S5.

## Discussion

Mean weekly recruitment was numerically higher during the intervention phase than during the control phase, but the Wilcoxon signed-rank test did not indicate a statistically significant difference. More importantly, the segmented interrupted time-series analysis showed neither a statistically significant immediate level change at CTRSS implementation nor a statistically significant post-implementation slope change. Taken together, these findings do not support a consistent pooled effect of CTRSS implementation on weekly recruitment.

Recruitment counts represented initial enrollments during the observation period and were not adjusted for subsequent withdrawals, as follow-up attrition was outside the scope of the recruitment outcome. Total enrollment ranged from 1 to 92 participants across trials; trials with no enrollment during the valid observation period were excluded according to the predefined eligibility criteria. Recruitment also varied considerably from week to week within trials, without a consistent temporal pattern across studies.

A persistent challenge in clinical trial recruitment is the limited time available to responsible staff, who are often simultaneously involved in patient care^19,20^. Fitzer et al. reported that recruitment activities may even interfere with patient care^5^. CTRSS were developed to address this issue by aiding in the identification of potential candidates for clinical trials, but the introduction of a new system represents a change-management process that requires an initial investment of staff time^21,22^.

Operational and compliance-related challenges were substantial. Reporting recruitment numbers and coordinating with study teams required considerable effort, while case-specific feedback on eligibility decisions and on which system suggestions led to enrollment remained incomplete despite repeated follow-up; some centers also discontinued participation. Consequently, trials that did not meet the predefined eligibility criteria and observation weeks without valid recruitment activity had to be excluded, reducing the effective sample size and statistical power. Because some inactive weeks may have coincided with implementation-related workload, particularly during rollout, their exclusion may have introduced selection bias and affected the estimated intervention effect in either direction. In a sensitivity analysis retaining weeks affected by staff absence, system downtime, or recruitment pauses, the intervention effect remained non-significant, whereas inactive weeks were associated with lower enrollment. These findings suggest that operational constraints and implementation demands—including training, technical coordination, and workflow adaptation—may have influenced recruitment performance.

Reports from participating study teams indicated that the interval between identifying a potentially eligible patient and enrollment varied considerably, ranging from hours to several months. Such variability may have attenuated short-term intervention effects in our weekly aggregate analyses, particularly when enrollment occurred substantially later than the initial CTRSS-based identification^23^. Exploratory lag analyses covering 1–4 weeks did not reveal a consistent delayed association between CTRSS implementation and enrollment. This may indicate that delays were highly heterogeneous, extended beyond the investigated lag period, or were insufficiently captured by aggregate weekly enrollment counts. Longer follow-up periods and patient-level attribution data would be required to assess identification-to-enrollment latency more directly.

These practical constraints mirror prior evidence. Earlier reviews of CTRSS reported heterogeneous methods and few clinical impact evaluations. Cuggia et al. reviewed 33 publications describing 28 patient-recruitment decision-support systems and concluded that methodological differences precluded a reliable overall assessment, although pre-screening appeared to be the most promising application^24^. Köpcke et al. reviewed 101 publications describing 79 systems; only five quantified effects on enrollment, and most publications focused primarily on technical performance^25^. More recent studies have likewise evaluated system design, retrieval accuracy, or technical feasibility, often retrospectively or outside routine clinical use, rather than effects on recruitment under real-world conditions^26–29^. Ni et al. evaluated automated pre-screening in six pediatric emergency-department trials over 12 months and reported an overall enrollment increase of 11.1%, although statistically significant effects were observed in only two trials^9,29^.

Consistent with the mixed literature, our multicenter evaluation showed substantial heterogeneity in recruitment patterns and estimated intervention effects. The recruIT implementation relied on routine data from heterogeneous source systems mapped to the OMOP CDM, resulting in site-specific differences in data availability, completeness, and timeliness. Technical staff therefore needed to understand both the common data model and the locally available data. Although this flexibility enabled adaptation to local conditions, as recommended by Blasini et al.^30^, it also allowed eligibility criteria to be implemented differently across sites, reducing standardization and comparability. In addition, limitations in data quality and query-engine expressiveness restricted the range of trials eligible for inclusion.

Higher criteria-revision values were associated with higher recruitment in some analyses, but this pattern was not consistent across trials or CTRSS types. This association should not be interpreted causally: revision activity may have served as a proxy for implementation maturity, local expertise, workflow integration, team engagement, or favorable operational conditions. Its overlap with the intervention phase, time since implementation, and increasing familiarity with the system further limits causal attribution. Nevertheless, the findings suggest that iterative refinement of computable cohort definitions may be more relevant than the initial technical activation of the CTRSS alone.

The segmented ITS design provided a stronger evaluation than a simple pre/post comparison because it distinguished between the baseline recruitment trend, an immediate implementation-associated level change, and a subsequent change in trend^15^. However, no suitable control outcome or non-equivalent dependent variable was available for the present evaluation. Together with the substantial heterogeneity across trials and sites, the absence of both an immediate level change and a post-implementation slope change is compatible with a context-dependent intervention whose effects may depend on local data availability, workflow integration, implementation fidelity, staff engagement, and downstream consent processes.

Ewusie et al. highlighted the limited methodological guidance regarding sample-size requirements for ITS studies^15^. Although recommendations regarding the number of observations and time points have been proposed for some ITS settings, no generally accepted threshold exists for the number of trials, clusters, or repeated observations required for segmented mixed-effects ITS models^31^. Accordingly, the present analysis may have been underpowered to detect small implementation effects, particularly given the limited number of trials, heterogeneous baseline recruitment rates, and unequal numbers of observations across trials.

This study has several limitations. The included trials differed in specialty, design, baseline recruitment rate, recruitment pathway, and the extent to which eligibility assessment could be represented using structured data. Such heterogeneity reduces comparability and limits the interpretability of a single pooled estimate. Exploratory subgroup and interaction analyses did not identify robust effect modification, but the small numbers within individual strata substantially restricted statistical power. These findings should therefore be considered hypothesis-generating.

Only one site used KAS+. Consequently, software platform and study site were perfectly confounded, and differences between KAS+ and recruIT cannot be attributed to the software systems independently of local implementation conditions.

Voluntary participation may also have introduced selection bias because participating teams may have differed from non-participating teams in motivation, technical readiness, available personnel, or perceived recruitment need. Some trials recruited hospitalized patients, whereas others contacted patients after discharge or during outpatient follow-up. These differences may have influenced both the likelihood of identifying eligible patients through a CTRSS and the probability that an identified patient was ultimately enrolled.

Exploratory subgroup, interaction, lag, and implementation-intensity analyses did not identify a robust or consistent pattern of effect modification or delayed intervention effects (Supplementary Tables S3 and S4a–c). The shift of the intervention estimate from above to below 1 when criteria revision was modeled jointly likely reflects collinearity between intervention phase, implementation maturity, and revision activity rather than evidence of a causal negative intervention effect. Given the limited number of trials within individual strata, these analyses should be interpreted as hypothesis-generating.

The primary interpretation remained unchanged in the sensitivity analysis restricted to the 26 trials with a single control-to-intervention transition and in the analysis accounting for inactive recruitment weeks.

A further limitation is that weekly enrollment represents a distal outcome of the recruitment process. The study could not determine whether patients identified by the CTRSS were reviewed, confirmed as eligible, approached, consented, or enrolled through another pathway. It also could not distinguish between patients who were ineligible, were not approached, declined participation, or were lost during later recruitment steps. Potential improvements in patient identification or screening efficiency may therefore not have translated into measurable differences in final enrollment. Patient-level process measures, including the numbers of patients identified, reviewed, confirmed as eligible, approached, consented, and enrolled, as well as the positive predictive value of CTRSS suggestions and the identification-to-enrollment interval, would allow a more direct assessment of the mechanisms through which CTRSS may influence recruitment.

Although the quantitative analyses did not demonstrate a statistically significant pooled effect, qualitative feedback was more favorable. In a sub-analysis, Stein et al. interviewed participants from three sites; 7 of 11 reported satisfaction with the system, and 8 of 11 indicated that they would use it again^18^. These findings indicate perceived usefulness among the interviewed participants even when quantitative effects on recruitment were limited.

The discrepancy between the quantitative and qualitative findings suggests that perceived usefulness and measurable recruitment effects represent different dimensions of implementation success. CTRSS may reduce screening workload, improve awareness of potentially eligible patients, or support recruitment workflows without necessarily producing an immediate or uniform increase in enrollment.

The present study did not demonstrate a statistically significant pooled effect of CTRSS implementation on weekly recruitment: neither an immediate change in recruitment level nor a gradual post-implementation change in trend was detected. Nevertheless, limited but generally favorable qualitative feedback suggests that CTRSS may provide process-related benefits in selected settings. Together with the exploratory association with criteria revisions, these findings suggest that CTRSSs may offer context-dependent benefits, particularly when computable eligibility criteria are iteratively refined and systems are well integrated into local workflows.

Future evaluations should include larger or more homogeneous trial samples, longer follow-up periods, prespecified subgroup analyses, patient-level process measures, and indicators of implementation fidelity. Process measures should capture the pathway from identification to enrollment, including whether CTRSS-identified patients were reviewed, confirmed as eligible, approached, consented, and ultimately enrolled. Attribution of enrollment to individual system suggestions and assessment of identification-to-enrollment latency would allow a more direct evaluation of underlying mechanisms. Compliance may be improved by providing participating sites with direct operational value, such as automated screening and enrollment reports. Where feasible, suitable comparison series or control outcomes should be incorporated, and technical performance, workflow integration, implementation quality, and recruitment outcomes should be evaluated jointly rather than relying solely on final enrollment counts.

## Methods

### Participating sites

This multicenter study was planned to be conducted at a total of ten different university hospitals, meaning that trials were included in the study at multiple consortium sites, but each trial recruited only locally at its respective site. The full scope of the study was described by Boeker et al.^32^. Four of the planned sites were excluded because of a lack of trials to include in the study or for other organizational reasons. Five of the final participating sites used the recruIT software and one used KAS+. Stakeholders were defined as all individuals involved in patient recruitment for clinical trials at the participating university hospitals. The principal investigator of each participating site was responsible for contacting the stakeholders within their hospital to ask if they would be willing to test the recruitment tool and participate in the study. The selection of individuals to approach was left to the PIs to make the best use of their expertise and network.

### Intervention

This controlled study was designed to test the use of a CTRSS to recruit subjects for clinical trials (intervention). During the intervention phase, one of the tools described below was used to assist the  study participants in recruiting patients.

RecruIT is a modular Clinical Trial Recruitment Support System (CTRSS) based on the OMOP CDM developed by Observational Health Data Sciences and Informatics (OHDSI). The OHDSI ATLAS software is used to define and check eligibility criteria^33^. A web-based screening list makes the search results available to trial staff. The modules can be customized to local conditions by selecting the information to be displayed on the screening list^17^.

The recruIT tool was used at five sites and adapted to local conditions using the available settings. At all sites, the EHR patient identification number was displayed as part of the screening list to enable trial staff to identify and locate patients in the hospital. Data were transferred from the primary clinical systems to OMOP CDM individually at each site.

The second tool evaluated is a CTRSS, which is integrated with a larger infrastructure called KAS+. The details of this system were previously described by Stein et al.^18^. The eligibility criteria were defined directly via the CentraXX user interface and the search query was executed daily. The proposals generated in this way were then summarized in a list, which was sent to the trial staff to be used within their usual pre-screening workflows. The trial staff then provided feedback on the proposals by transferring back the annotated lists to the local study group.

### Study design

In order to encourage trial centers to participate in the study, despite the limited staff time, the primary argument was a potential increase in recruitment numbers or a time saving through the digitized pre-screening process. Consequently, a control group-based study design was deemed unfeasible, as we anticipated a high rate of attrition in the control group. Given the multicenter nature of the study and the logistical complexities of coordinating across several sites, a cluster-randomized design would have been resource-intensive and challenging to implement. Instead, an Interrupted Time Series (ITS) design was chosen, which allowed us to assess the real-world impact of the intervention using existing data across multiple centers, without the need for traditional randomization, by collecting data before and after the intervention to analyze trends and evaluate long-term effects^34,35^. The primary analysis used segmented ITS regression to estimate an immediate level change at implementation and a change in post-implementation slope.

Each trial required an observation period of at least 6 months (beginning with the inclusion of the trial into the evaluation study), divided into a period before use of the recruiting tool (control phase) and a period after (intervention phase), with at least one month in each phase. The exact start time of the intervention was randomized. The study was conducted between 2021-01-01 and 2022-10-31 (closure of the data set)—the observation phase could be started at any time up to 2022-04-30 at the latest. After the intervention phase, the study participants were free to continue using the tool. There was no termination phase.

In the following sections, the descriptions are oriented towards the data and questionnaires that exert an influence on the quantitative results of the present study. The remaining data collection instruments have been described in the published study protocol by Boeker et al.^32^.

### Data collection

Participants were given questionnaires to complete at various points throughout the study to capture key information about the trials. At the beginning, important trial information (type, registration information as unique trial identifier), as well as department, start, and end of recruitment, was recorded. During the intervention period, we documented the number of enrollments per week for each participating trial, as well as any influencing staff  absences and system downtimes. Also, we documented any adjustments of the eligibility criteria or other configuration of the tool (criteria revisions). The number of criteria revisions was recorded as an ordinal update counter (0–4) of the computable cohort definition/CTRSS configuration; 0 indicates that no update had been implemented/documented yet, and 1–4 denote successive revisions during the intervention phase.

We used the REDCap software (version 11.4.0) to create, record, and store all questionnaires^36^.

Filter criteria were established to search the database (OMOP/KAS+ database) based on the inclusion and exclusion criteria of the participating trials. It is important to note that these criteria may differ from the official eligibility criteria described in the trial protocols. Eligibility criteria include information on patients that is collected regularly, as well as information collected during an additional appointment. Since recruIT and KAS+ are pre-screening systems, they often use fewer and more general criteria. Examples include smoking status, which is often not documented, and willingness to use appropriate contraceptive methods for the duration of the study. Furthermore, some criteria must be assessed by medical staff, for which a technical definition cannot be found. One example is the assessment of whether other illnesses prevent participation in the study. In order to establish comparability between trials, the filter criteria were categorized and analyzed. The analysis was conducted by one author to ensure comparability across all trials^37^.

### Inclusion of trials

We only included trials that actively (and prospectively) recruited participants for a total of at least three weeks during the control and intervention periods. Active recruiting was defined as a period during which there was neither a system failure nor key users off-site for more than 2 days of a working week. Second, we only considered trials that recruited at least one participant during the whole valid observation time. A proportion of the trials were multicenter in nature; however, each trial was included only once in the evaluation study.

Pauses in recruitment due to staff shortages or other extraneous factors were explicitly documented. During the final evaluation period, weeks during which recruitment did not take place were excluded.

### Data analysis

Data on the intervention impact was first analyzed descriptively: the Kolmogorov-Smirnov^38^ test was used to test normality, and it indicated that the data distribution  deviated substantially from a normal distribution.

Wilcoxon signed-rank tests^39,40^ were therefore used to compare statistics for the weekly recruitments observed during the control and intervention phases. *P* < 0.05 was considered statistically significant. Valid trials were included in a generalized linear mixed model analysis of weekly recruitment counts. The intervention indicator (control vs. intervention phase) was included as a fixed effect, and the clinical trial was included as a random effect.

To account for potential overdispersion, a negative binomial distribution was applied for the mixed model. Models with random intercept only or random intercept and slope, allowing a different effect of the intervention for each clinical trial, were compared using Akaike’s information criterion. We calculated the incidence rate ratio for the effect of intervention on the recruiting numbers per week (crude model A) with a (asymmetric) two-tailed 95% confidence interval using the Wald method. In a second model (model B), we added the variable criteria revision as a fixed effect to investigate the effect of iterative refinement of cohort definitions during the usage of the CTRSS. The criteria revision number was included as an ordinal covariate.

A two-sided 95% confidence interval for the incidence rate ratio associated with criteria revisions was also calculated using the Wald method. A two-tailed significance level of 5% was considered statistically significant. Residuals were tested for potential violations of the assumed distribution.

### Segmented ITS analysis

Weekly enrollments were modeled using segmented interrupted time-series regression with a negative binomial distribution and log link. For trial *i* in week *t*, let *Y*_it_ denote the number of enrollments. We specified:

*Y*_it_ ~ NegBin(*μ*_it_, θ), log(*μ*_it_) = *β*_0_ + *β*_1_·*time*_it_ + *β*_2_·*post*_it_ + *β*_3_·*time_after*_it_ + *u*_0i_ + *u*_1i_·*post*_it_

Here, *time*_it_ is the continuous time index (weeks since the start of observation for trial *i*); post_it_ is an indicator for the implementation period (0 before go-live, 1 at/after go-live) capturing an immediate level change; and time_after_it_ is the time since implementation (0 before go-live, and time_it_ − *t0*_i_ + 1 at/after go-live, where *t0*_i_ is the first week of implementation for trial *i*), capturing a slope change relative to the pre-implementation trend. For each trial, the first documented CTRSS go-live was used as the interruption point. Twenty-six of the 29 trials showed a single clean transition from the control to the intervention phase, whereas three trials showed multiple transitions. To assess the robustness of the ITS estimates, we performed a sensitivity analysis restricting the segmented ITS model to the 26 single-switch trials.

Trial-specific random effects *u*_0i_ (random intercept) and *u*_1i_ (random slope for the level change) were included to account for between-trial heterogeneity. Exponentiated coefficients are reported as incidence rate ratios (IRR) with two-sided 95% confidence intervals: exp(*β*_2_) represents the level change and exp(*β*_3_) the post-implementation change in slope^41^.

As a sensitivity analysis, we re-estimated Models A and B including weeks that were previously excluded due to staff absence, system downtime, or recruitment pauses. These weeks were retained and adjusted for using an indicator covariate (inactive_week), defined based on the documented downtime/absence variables, to assess whether excluding inactive weeks biased the intervention or criteria revision estimates. Inactive_week was coded as 1 for weeks with >2 days of key-user absence and/or documented system downtime and/or recruitment stop, and 0 otherwise^41^.

To explore whether CTRSS effects might be delayed, we performed exploratory lag analyses using 1–4 week lags of the intervention indicator and of the criteria revision covariate. Lagged terms were defined at the trial level and were included alongside the concurrent (non-lagged) term to assess short-term delayed associations with weekly enrollments. In addition, we explored heterogeneity by implementation intensity by stratifying trials according to the maximum documented number of criteria revisions during the intervention phase (max number of criteria revisions = 0 vs. ≥1). Within each stratum, we re-estimated Model A (intervention only) and Model B (intervention + criteria revision). All lag and intensity-stratified analyses were considered hypothesis-generating^41^.

Calculations were done using Python 3.12^42^ and R 4.1.3^43^, including the glmmTMB package^44^. After data collection, questionnaires and surveys were exported from REDCap and analyzed and processed using RStudio software^45^.

### Data protection

Pseudonymized data are processed under individual site data protection laws for medical care and research. The evaluation study does not use any trial participant data. To conduct the study, a data protection concept was drawn up at all locations and discussed with the local data protection officers at the hospitals. Patient identification using pseudonymized data via hospital information systems or by contacting treating physicians adheres to data protection regulations through hospital access controls and cooperation agreements. Original data remain with their respective locations and are processed according to data protection and ethics committee votes. Personal data of the patients concerned were only processed within the hospitals. No personal data were processed for the evaluation within this study, only key figures on the recruitment numbers of the included trials.

### Ethics approval and consent to participate

This study was approved by the ethics review committee at the Friedrich-Alexander-Universität Erlangen-Nürnberg (approval number 89_20 B). This vote was used as a template for all other participating sites. All sites obtained a corresponding vote from their local ethics committee:

- Ethics Committee at the Technical University of Dresden (SR-EK-411092020)
- Ethics Committee of the University of Freiburg (290/20)
- Ethics Committee of the Faculty of Medicine at Goethe University c/o University Hospital (20-756)
- Ethics Committee of the Department of Medicine Justus-Liebig University Giessen (193/20)
- University Medicine Greifswald Ethics Committee (BB 084/20)
- Ethics Committee II of the University of Heidelberg Medical Faculty Mannheim (2020-624 N)
- Ethics Committee of the Faculty of Human Medicine of the Philipps University of Marburg / University Hospital Giessen and Marburg GmbH - Marburg site (87/20)
- Ethics Committee of the Otto von Guericke University at the Medical Faculty and the University Hospital Magdeburg A.ö.R. (115/20)

## Supplementary information

Supplementary_IT-Supported Patient Recruitment. Platform Evaluation and Lessons Learned.

Supplementary Information

## Data Availability

All data generated or analyzed during this study are included in this published article or published under https://doi.org/10.5281/zenodo.15117924.

## Code availability

All used analysis code is available under https://doi.org/10.5281/zenodo.15117924.
