# Supplementary material for: Platform evaluation and lessons learned with IT-supported patient recruitment
Source: NPJ Digit Med. 2026 Oct 30;9:828. doi: 10.1038/s41746-026-03366-y (PMC13722682; doi:10.1038/s41746-026-03366-y)
Supplement: Supplementary file 1 [file 41746_2026_3366_MOESM1_ESM.pdf]

Table S1

Trial	Site	CTRSS	Total weeks	Control weeks	Intervention weeks	Total recruited (studyperiod)	Max criteria revision	# updates (max criteria revision)	Weeks v0	Weeks v1	Weeks v2	Weeks v3	Weeks v4
1	6	KAS+	66	26	40	49	1	1	34	32	0	0	0
2	6	KAS+	52	9	43	4	0	0	52	0	0	0	0
3	6	KAS+	60	25	35	3	1	1	26	34	0	0	0
4	6	KAS+	44	11	33	95	4	4	12	1	1	9	21
47	10	recruIT	30	7	23	18	2	2	13	4	13	0	0
49	10	recruIT	30	10	20	3	1	1	15	15	0	0	0
50	10	recruIT	30	10	20	15	2	2	13	4	13	0	0
52	10	recruIT	30	20	10	4	0	0	30	0	0	0	0
53	10	recruIT	30	10	20	2	3	3	13	3	1	13	0
54	10	recruIT	30	5	25	2	1	1	13	17	0	0	0
56	10	recruIT	30	10	20	1	1	1	14	16	0	0	0
8	2	recruIT	36	26	10	8	0	0	36	0	0	0	0
9	2	recruIT	36	14	22	84	0	0	36	0	0	0	0
11	2	recruIT	36	18	18	16	0	0	36	0	0	0	0
12	2	recruIT	36	22	14	4	0	0	36	0	0	0	0
16	2	recruIT	36	23	13	3	0	0	36	0	0	0	0
17	2	recruIT	35	13	22	6	0	0	35	0	0	0	0
18	2	recruIT	36	14	22	2	0	0	36	0	0	0	0
24	2	recruIT	22	13	9	4	0	0	22	0	0	0	0
25	2	recruIT	22	8	14	2	0	0	22	0	0	0	0
58	3	recruIT	15	9	6	2	0	0	15	0	0	0	0
6	5	recruIT	48	15	33	3	0	0	48	0	0	0	0
7	5	recruIT	39	30	9	27	0	0	39	0	0	0	0
27	5	recruIT	42	11	31	2	0	0	42	0	0	0	0
28	5	recruIT	39	24	15	2	0	0	39	0	0	0	0
29	5	recruIT	39	11	28	1	0	0	39	0	0	0	0

32	1	recruIT	27	15	12	2	0	0	27	0	0	0	0
37	1	recruIT	27	22	5	3	0	0	27	0	0	0	0
38	1	recruIT	27	14	13	62	0	0	27	0	0	0	0

Table S2

**Group**	**criteria revision**	**Relative expected weekly enrollment (vs v0)**	**Lower 95% CI**	**Upper 95% CI**
All sites	0	1	1	1
All sites	1	1.601	1.143	2.242
All sites	2	2.563	1.306	5.027
All sites	3	4.104	1.493	11.27
All sites	4	6.57	1.707	25.266
recruIT sites	0	1	1	1
recruIT sites	1	1.161	0.627	2.152
recruIT sites	2	1.348	0.393	4.631
recruIT sites	3	1.565	0.246	9.966
recruIT sites	4	1.817	0.155	21.447
KAS+ site	0	1	1	1
KAS+ site	1	2.151	1.784	2.593
KAS+ site	2	4.627	3.183	6.724
KAS+ site	3	9.952	5.678	17.434
KAS+ site	4	21.407	10.129	45.207

Table S3

Section	Studies	Weeks	Model	Intervention_est	Intervention_p	criteria revision_est	criteria revision_p	EffectMod_var	EffectMod_est	EffectMod_p	Intervention_x_var_est	Intervention_x_var_p
Overall	24	707	A - crude	0.4325	0.219							
Overall	24	707	B - with criteria revision	0.1513	0.66998	0.4367	0.00549					
Effect modification: n_krit_positiv	17	561	Fixed effects (no interaction)	0.41325	0.307			n_krit_positiv	-0.04703	0.853		
Effect modification: n_krit_positiv	17	561	With interaction	1.9935	0.0307			n_krit_positiv	0.1511	0.54	-0.4843	0.0637
Effect modification: n_krit_neg	17	561	Fixed effects (no interaction)	0.3543	0.362			n_krit_neg	0.2302	0.0284		
Effect modification: n_krit_neg	17	561	With interaction	0.39043	0.4648			n_krit_neg	0.23443	0.0386	0.01301	0.9215
Effect modification: n_krit_all (sum)	17	561	Fixed effects (no interaction)	0.33512	0.393906			n_krit_all	0.15813	0.086844		
Effect modification: n_krit_all (sum)	17	561	With interaction	0.81999	0.275248			n_krit_all	0.17938	0.056728	-0.07971	0.453381
Specialty (exploratory)	24	707	Specialty interaction model					medical_department				
Specialty (exploratory)	12	346	Neurology stratum	0.1465	0.689401							

Table S4a Lag models

Group	criteria revision	Relative expected weekly enrollment (vs v0)	Lower 95% CI	Upper 95% CI
All sites	0	1	1	1
All sites	1	1.601	1.143	2.242
All sites	2	2.563	1.306	5.027
All sites	3	4.104	1.493	11.27
All sites	4	6.57	1.707	25.266

recruIT sites	0	1	1	1
recruIT sites	1	1.161	0.627	2.152
recruIT sites	2	1.348	0.393	4.631
recruIT sites	3	1.565	0.246	9.966
recruIT sites	4	1.817	0.155	21.447
KAS+ site	0	1	1	1
KAS+ site	1	2.151	1.784	2.593
KAS+ site	2	4.627	3.183	6.724
KAS+ site	3	9.952	5.678	17.434
KAS+ site	4	21.407	10.129	45.207

Table S4b Intensity strata

**Stratum**	**Model**	**Term**	**# trials**	**# weeks**	**IRR (95% CI)**	**p-value**	**Note**
Max criteria revision = 0 (no updates)	Model A (crude)	Intervention	20	538	1.487 (0.607–3.643)	0.385	Model B not estimable (criteria revision constant 0).
Max criteria revision ≥1 (updates)	Model A (crude)	Intervention	9	276	1.606 (0.358–7.193)	0.536	
Max criteria revision ≥1 (updates)	Model B (+criteria revision)	Intervention	9	276	0.413 (0.178–0.957)	0.039	
Max criteria revision ≥1 (updates)	Model B (+criteria revision)	criteria revision	9	276	2.302 (1.948–2.720)	<0.001	

Table S4c Strata definition

**trial**	**n_control**	**n_intervention**	**total_recruited**	**n_weeks**	**max_criteria revision**	**Stratum**	
2	9	27	3	36	0	Max criteria revision = 0 (no updates)	
6	15	24	2	39	0	Max criteria revision = 0 (no updates)	
7	25	6	24	31	0	Max criteria revision = 0 (no updates)	
8	22	6	5	28	0	Max criteria revision = 0 (no updates)	
9	14	17	82	31	0	Max criteria revision = 0 (no updates)	

11	17	13	15	30	0	Max criteria revision = 0 (no updates)
12	19	12	4	31	0	Max criteria revision = 0 (no updates)
16	20	8	3	28	0	Max criteria revision = 0 (no updates)
17	13	14	6	27	0	Max criteria revision = 0 (no updates)
18	14	14	2	28	0	Max criteria revision = 0 (no updates)
24	10	3	4	13	0	Max criteria revision = 0 (no updates)
25	7	10	1	17	0	Max criteria revision = 0 (no updates)
27	11	14	2	25	0	Max criteria revision = 0 (no updates)
28	22	12	2	34	0	Max criteria revision = 0 (no updates)
29	11	12	1	23	0	Max criteria revision = 0 (no updates)
32	15	12	2	27	0	Max criteria revision = 0 (no updates)
37	22	4	3	26	0	Max criteria revision = 0 (no updates)
38	13	11	58	24	0	Max criteria revision = 0 (no updates)
52	20	8	4	28	0	Max criteria revision = 0 (no updates)
58	9	3	2	12	0	Max criteria revision = 0 (no updates)
1	26	37	49	63	1	Max criteria revision ≥1 (updates)
3	25	28	3	53	1	Max criteria revision ≥1 (updates)
4	11	31	92	42	4	Max criteria revision ≥1 (updates)
47	7	11	11	18	2	Max criteria revision ≥1 (updates)
49	10	11	3	21	1	Max criteria revision ≥1 (updates)
50	10	11	9	21	2	Max criteria revision ≥1 (updates)
53	10	11	1	21	3	Max criteria revision ≥1 (updates)
54	5	11	2	16	1	Max criteria revision ≥1 (updates)
56	10	11	1	21	1	Max criteria revision ≥1 (updates)

Table S5 IRR summary (sensitivity_inactive_weeks)

term	beta	IRR	CI_low	CI_high	p	model
intervention	0.466072	1.593721	0.743368	3.416808	0.230987	A primary (inactive excluded)
intervention	0.156948	1.169935	0.5063	2.703431	0.713416	B primary (inactive excluded)
criteria revision	0.465707	1.59314	1.034974	2.452327	0.034327	B primary (inactive excluded)
intervention	0.483697	1.62206	0.796034	3.305234	0.182899	A sensitivity (inactive included + indicator)
inactive_week	-0.74794	0.473341	0.279538	0.801507	0.005379	A sensitivity (inactive included + indicator)
intervention	0.224462	1.251649	0.578992	2.705778	0.568224	B sensitivity (inactive included + indicator)
criteria revision	0.409513	1.506084	0.943816	2.403319	0.08589	B sensitivity (inactive included + indicator)
inactive_week	-0.70708	0.49308	0.281056	0.865052	0.013683	B sensitivity (inactive included + indicator)

Predictor	Lag (weeks)	# trials	# weeks	IRR (95% CI)	p-value
Intervention (lagged)	1	29	785	0.794 (0.337–1.867)	0.596
Intervention (lagged)	2	29	756	1.167 (0.497–2.737)	0.723
Intervention (lagged)	3	29	727	1.218 (0.514–2.889)	0.654
Intervention (lagged)	4	29	698	1.233 (0.543–2.799)	0.616
criteria revision (lagged)	1	29	785	0.948 (0.590–1.523)	0.824
criteria revision (lagged)	2	29	756	0.950 (0.727–1.240)	0.704
criteria revision (lagged)	3	29	727	0.898 (0.737–1.094)	0.285
criteria revision (lagged)	4	29	698	0.842 (0.705–1.004)	0.056

Valid trials (sensitivity_inactive_weeks)

trial	n_control	n_intervention	total_recruited	n_weeks
1	26	39	49	65
2	9	42	4	51
3	25	34	3	59
4	11	32	92	43
6	15	32	3	47
7	30	9	27	39
8	26	9	7	35

9	14	22	84	36
11	18	18	16	36
12	22	14	4	36
16	23	12	3	35
17	13	22	6	35
18	14	22	2	36
24	13	9	4	22
25	8	13	2	21
27	11	31	2	42
28	24	15	2	39
29	11	28	1	39
32	15	12	2	27
37	22	4	3	26
38	14	13	62	27
47	7	22	16	29
49	10	19	3	29
50	10	19	15	29
52	20	9	4	29
53	10	19	2	29
54	5	24	2	29
56	10	19	1	29
58	9	6	2	15

**Stratum**	**Model**	**Term**	**# trials**	**# weeks**	**IRR (95% CI)**	**p-value**	**Note**
Max criteria revision = 0 (no updates)	Model A (crude)	Intervention	20	538	1.487 (0.607–3.643)	0.385	Model B not estimable (criteria revision constant 0).
Max criteria revision ≥1 (updates)	Model A (crude)	Intervention	9	276	1.606 (0.358–7.193)	0.536	
Max criteria revision ≥1 (updates)	Model B (+criteria revision)	Intervention	9	276	0.413 (0.178–0.957)	0.039	
Max criteria revision ≥1 (updates)	Model B (+criteria revision)	criteria revision	9	276	2.302 (1.948–2.720)	<0.001	

Counts (sensitivity_inactive_weeks

n_rows_primary	n_rows_sens	n_trials
780	1014	29

Table S6 single-switch ITS sensitivity

Model component	IRR	95% CI	p-value
time	0.970	0.914–1.031	0.330
post	1.921	0.657–5.611	0.233
time_after	1.039	0.969–1.114	0.282
